# Walking the Talk: Adopting and Adapting Sustainable Scientific Software Development processes in a Small Biology Lab

**DOI:** 10.5334/jors.35

**Published:** 2016-11-29

**Authors:** Michael R. Crusoe, C. Titus Brown

**Affiliations:** 1 Microbiology and Molecular Genetics, Michigan State University, East Lansing, MI, USA; 2 Computer Science and Engineering, Michigan State University, East Lansing, MI, USA

**Keywords:** WSSSPE, k-mer

## Abstract

The khmer software project provides both research and production functionality for largescale nucleic-acid sequence analysis. The software implements several novel data structures and algorithms that perform data pre-fltering for common bioinformatics tasks, including sequence mapping and *de novo* assembly. Development is driven by a small lab with one full-time developer (MRC), as well as several graduate students and a professor (CTB) who contribute regularly to research features. Here we describe our efforts to bring better design, testing, and more open development to the khmer software project as of version 1.1. The khmer software is developed openly at http://github.com/dib-lab/khmer/.

## (1) Introduction

Computational tools for analyzing large volumes of DNA/RNA sequencing data have become increasingly necessary over the last decade. The growth of sequencing capacity and the associated expansion of scientific problems being studied with sequencing is driving the rapid development of many new tools, both for handling data on large scales and to address new and different biological problems.

The khmer software was born from a need to more scalably analyze short fixed-length (20–30 character) words, or “k-mers”, in large DNA sequencing data sets. The use of k-mers in DNA sequence analysis is common because they can be easily hashed, counted, and compared within and between data sets. However, as data sets have grown in size, approaches to analyzing k-mers have fallen behind the memory and compute scaling curves. khmer provides several functions: approximate k-mer counting using a CountMin Sketch [10], an implementation of a compressible k-mer connectivity graph [8], and a streaming lossy compression algorithm for large data sets [2]. These were first implemented as a part of bioinformatics research publications, but due to their broad utility have now been used in several hundred data analysis publications.

We developed the khmer software as an open source project since the beginning: the software is under the BSD license, and we use GitHub for most development activities, including co-ordinating contributions, performing code review, and tagging releases. We provide a wide variety of tutorials and user documentation, both as part of the khmer project itself and also as part of a range of workshop material. Adoption of khmer is driven not only by its utility in addressing otherwise difficult or intractable problems, but also by CTB's blogging, research preprints and publications, and presentations.

The user base for khmer is unknown but appears to be significant. While we do not track users per se, there are over 1500 downloads of khmer a month from the Python packaging distribution site, and about 2000 visits to the khmer documentation site a month. The GitHub site is in the 97th percentile of software on GitHub for both ‘stars’ (129) and ‘forks’ (76), indicating general interest. Scientifically, there are over 30 papers citing khmer for data analysis purposes, and the algorithms and approaches initially implemented in khmer have been adapted to and incorporated in several other software packages.

The main challenge for us in developing khmer has been to build a stable and reliable software project while simultaneously supporting an energetic research program in bioinformatics. This has traditionally been hard for small scientific labs due to many factors including lack of expertise and lack of sustained funding. Below, we discuss our experience in navigating the challenges in making a small-lab software project sustainable. We focus particularly on how we changed our software development process to support a more sustainable development process.

## (2) Background

khmer grew out of specific analysis needs, and was developed primarily on startup funding and as part of a USDA grant. Its development has led to at least two additional grants including the NIH BIG DATA grant that supported MRC [1]. Over its lifetime khmer has had 15 different contributors, with five currently active. The code consists of approximately 12.2k lines of C and C++ code, with scripts and tests written in Python (6.6k lines of code).

The software was initially written by CTB for other purposes during his graduate work at Caltech, and then extended so far as to be almost entirely rewritten for research in his faculty position at Michigan State University. By July of 2013, when MRC started, the software had its current level of functionality, but we faced a number of specific challenges.

1. We had no formal development model: there was no code review, no formatting requirements, no continuous integration, and no API stability requirements. As a result we were constantly in a state of uncertainty about khmer's quality and stability. In practice, this manifested as highly variable code quality, uneven density of bugs in different pieces of core functionality, and periods where key pieces of functionality did not function properly.
2. Our developers had a variety of experience: some were expert computational biologists with little to no programming experience, while others were experienced open source software developers with little to no computational biology background. This meant that we could not confidently rely on good domain understanding *and* good software development hygiene from any one developer. One particular outcome of this mismatch was the development of a significant ancillary codebase of redundant and semi-functional scripts that made use of core khmer functionality but was not integrated into the project; we also encountered situations where biologically inappropriate data transformations were made for sound engineering reasons, e.g. the elimination of ambiguous nucleo-tides from input data.
3. Like many bioinformatics projects, khmer is both *research* and *production* software: our lab is constantly extending khmer in new directions, at the same time as we and others apply its existing functionality to analyze biological data. While managing regular change is a traditional challenge for software development on any long-running project, the problem was exacerbated here: long-term planning was impractical given the high rate of technical innovation in sequencing data generation.
4. khmer exists within an ecosystem of tools. khmer itself primarily filters sequence data, which is generated in specific formats by upstream tools and is then consumed in those same formats by downstream tools. We had no systematic testing of khmer within its larger ecosystem, and generally relied on users to find problems. In one particular instance, a minor typo in a downstream processing output function meant that while all internal tests passed, no external programs could consume khmer output successfully.

Collectively these challenges made us believe that khmer software development was not sustainable without significant investment in software engineering. Either (1) the research development would falter in the face of increasingly high maintenance demands, or (2) khmer's stable functionality would start to deteriorate, or (3) both. To address these challenges, CTB secured three years of NIH funding through the 2012 NIH/NSF BIG DATA funding call, and hired MRC, a software developer with biology education and bioinformatics experience.

## (3) Upgrading the development process

### 3.1 The khmer lifecycle

As described above, khmer started as a small single-developer project but was never published, and development lapsed for several years. In 2010, we repurposed khmer as a testbed for trying out approaches to memory-efficient k-mer counting in large data sets [10]. Over the next few years, several developers contributed to khmer functionality, culminating in implementations of a compressible graph representation for DNA sequences and a streaming lossy compression algorithm [8] [2]. In addition to providing a demonstration implementation for the purposes of publishing these methods, khmer also proved directly useful in data analysis [6]. Because we provided khmer as open source software and discussed it online in social media, it was also adopted by a number of other groups who had similar problems.

As khmer was being used both as a methods testbed and for actual data analysis, the project lead (CTB) made a concerted effort to keep khmer extensible while maintaining existing functionality. This was largely reflected in a conservative approach to merging in contributions from graduate students in the lab, but was also enabled by a significant enthusiasm for automated tests at the unit, functional, and scripting level. Nonetheless during this period the software regularly suffered significant bugs, and large portions of the code base were added on a trial basis but then left unused when research went in different directions.

In 2013, significant funding for further software development was obtained through an NIH R01 grant, and MRC was hired to manage the development process. Also during this period, a number of new graduate students also joined the lab, and it became clear that they would be working on the khmer code base as part of their research. This made us take a step back to evaluate our overall process.

### 3.2 Evaluating the project's sustainability

To guide our development of a rational software development process, we applied the Software Sustainability Institute's Criteria-based assessment checklist [7] to the khmer project in September 2013 and shared the results with the community [4]. The summary from that report was grim: khmer met 19 of 44 (or 43%) of of the SSI's criteria for Usability, and 43 of 118 (or 36%) of the criteria for Sustainability & Maintainability, for an overall fulfillment of 62 of the 163 items, or 38%.

### 3.3 Changing our development process

For version 1.0, we adopted continuous integration, semantic versioning, acceptance testing, development standards, code coverage analysis, explicit citation information, and code review, among other process alterations and features. While these are standard software development and engineering practices outside of academia, we find that many scientific software developers are unaware of them. Moreover, their interaction with research goals has not been well explored, so we discuss them in more detail.

#### 3.3.1 Development standards and semantic versioning

We instituted a number of development standards, including coding styles and versioning requirements for backwards compatibility. Our goal was to have explicit written requirements that would inform new contributors of our expectations, whether they have significant prior programming experience or not. A particularly important part of this goal was to make sure that new contributors *within the lab* had a clear set of expectations.

Uniformity of coding styles helps maintain code readability and enables easier code review, so we chose a coding style standard for both C++ and Python. The specific choice for coding style was made somewhat arbitrarily, largely to avoid protracted bikeshedding discussions: the important goal was to have *some* coding standard. For C++, we chose the “One True Brace Style” and the Artistic Style program for indentation and bracing. For Python, we settled on the default PEP8 standard, for which several checking and reformatting tools exist.

We also imposed a backwards compatibility requirement on our command line scripts. While we did not want to stabilize the Python or C++ API because we are actively changing khmer internals, we felt that our command line scripts were sufficiently stable to require that there would never be any backwards-incompatible changes in subsequent releases on the 1.0 series.

We have therefore committed to *semantic versioning* [9] for the command line scripts that come with khmer. This imposes a three-tiered versioning system: for patch version number changes (khmer v1.0.x), only minor bug fixes and documentation updates are allowed; for minor version number changes (khmer v1.x), backwards compatibility of the command line scripts must be maintained; and, should we choose to break backwards compatibility, we would need to make a major version number change (khmer v2).

The importance of semantic versioning is that it allows developers, documentation writers, sysadmins, and package managers to predict the specific behavior from a range of versions, and to easily determine whether or not they should upgrade their installation. Of particular importance, pipeline developers and users can rely on stable behavior from minor releases. We expect this to make khmer a more reliable member of the sequencing analysis software ecosystem, and also reduce the confusion that existing users will experience with new releases of the software.

#### 3.3.2 Continuous integration

Continuous integration ensures that automated tests are executed regularly on standard platforms. While developers are expected to commit code with no failing tests, often they do not have convenient access to all of the supported platforms and installation environments. Continuous integration frees individual developers from having to execute their tests manually across many environments by automating the entire process on commit. Our continuous integration system, built on top of Jenkins and running on a Rackspace donated Linux server and an in-house Mac OS X machine, also runs style checkers and reports code coverage summaries.

The most important application of continuous integration for us has been automated checking of merge requests prior to code review or merge into the mainline. This automatically ensures a basic quality of committed code and also alerts developers to any platform incompatibilities before they merge. It also serves as an important check for less experienced software developers, who may have forgotten to run one or another element of the required checks on their contribution.

#### 3.3.3 Integrated code coverage analysis

Code coverage analysis is an important part of software development: statement coverage, or how many lines of code are executed in some way by unit and functional tests, can readily identify untested regions of code. Note that while executed code is not guaranteed to be correct, code that is not executed by tests is certainly not tested, so high code coverage is a necessary but not sufficient condition for thorough testing. While khmer had several hundred tests by the time MRC started working with it, the tests were all at the Python level and we had no estimate of how well they covered the C++ code base.

Combined C++ and Python code coverage was instituted in October 2013 and we were pleasantly surprised to find that over 80% of the khmer codebase was executed in the tests. Since October we have increased the code coverage percentage to over 90%. This number is now calculated on every pull request (see below) and significant decreases are flagged as “unhealthy” in our continuous integration system.

#### 3.3.4 Code contribution process and code review

While code review is an important part of ensuring that only “good” code and feature are included in a project, it is typically very time consuming to do systematic code reviews. In order to scale our development process to more contributors while enabling code review, we adopted the “GitHub Flow” model of code review [3]. In this model, changes are developed on an independent branch of code; this branch of code is linked to the main development repository via a “pull request”, which is an ongoing summary of changes together with free text discussion. When the developer feels that the changes are ready to be merged, they request a formal review, for which we have instituted a checklist; this checklist includes test coverage and coding style analysis, documentation review, and compatibility checking.

Our expectation is that this more formal but still lightweight development process will encourage contributions and also serve as a training and education process for less experienced developers. By making our developer contribution requirements explicit, we may also serve as a guide for other bioinformatics software projects.

#### 3.3.5 Integration and acceptance testing

An ongoing concern for khmer is how well our software integrates with other packages. Because khmer primarily consumes the output of upstream software, and the output of khmer is then fed into downstream software, we need to take into account a larger software ecosystem. Unfortunately, there are few real data format standards in this area: the sequencing companies that generate the source data are notoriously quick to change their output formats in arbitrary ways, and developers of other packages may introduce format changes intentionally (through feature extension) or unintentionally (through bugs). Standardization itself is probably a futile approach: while we expect A, C, G, and T to remain the primary characters in DNA sequence representations, the formats for data uncertainty and annotation evolve with sequencing technology, which in turn is changing quickly.

We therefore have instituted *acceptance testing* to ensure that khmer works with at least some upstream and downstream software packages. Our acceptance tests for khmer 1.0 take a subset of data through quality control, error trimming, digital normalization, and assembly; at the end we check that we obtain approximately the expected results, vice minor details that change with different versions of external software. We have been greatly aided in developing acceptance tests by our own standard “protocols” for sequence analysis: our acceptance tests go through the first three parts of the Eel Pond mRNAseq protocol (http://khmerprotocols.readthedocs.org/).

Acceptance testing proved to be extremely important in the release process. Four different bugs having to do with installation and command-line parameter handling were discovered in the 48 hours before release of version 1.0; these bugs generally had to do with common command line cases that were not readily testable at the unit and functional level.

We are also targeting our acceptance tests for Ubuntu 14.04, a Long Term Support version of Linux that will be supported through 2019. This should further decrease maintenance efforts for our acceptance tests.

#### 3.3.6 Citation information

Scientific funding for software maintenance depends on demonstrating the scientific utility of software; this is typically done via citations. For both algorithms and software, citations demonstrate usage, utility, and impact. However, scientific software may contain multiple novel algorithms, and the software itself may be published separately from the proof of concept of the algorithms. For khmer, this is a serious concern: we have publications or preprints on three novel approaches implemented in khmer, and we are also continually updating the software itself. We also have a significant online presence. This demonstrably confuses users: we have observed citations of the incorrect paper for the algorithm being used, citations of our documentation, and (oddly enough) citations of khmer documentation hosted on other institutions' Web sites.

To clarify and regularize citation practices, we added explicit citation guidelines in two places: first, in the CITATION file at the top of the distribution, and second, in the output from every script. We now ask that users cite not only the software itself (via a software paper), but also the algorithm papers relevant to the software features being used.

While we worry about appearing to be “citation greedy” we also believe quite strongly that our ongoing efforts to maintain the software are a critical part of our research, and that the researchers and developers involved in that effort should be acknowledged appropriately in the scientific literature. This can really only be addressed by requiring citation of the relevant software paper, which will be updated for every significant version release with contributor names. At the same time, we also believe that our algorithm contributions are independently important and should be acknowledged by citations. Hence, the requirement that when our algorithms are used, the relevant algorithms paper should be cited.

### 3.4 Releasing version 1.0

On April 1st, 2014, we released khmer 1.0. While by no means a finished product, we now believe we are on a much more sustainable development path. In particular, khmer now meets 69% of the Software Sustainability Institute's checklist [5].

Some of the criteria that wasn't being initially met but now are fulfilled are: (A) Comprehensive documentation (of the scripts). (B) The documentation lists what version it applies to (C) An automated build system (D) Documentation of the build (E) Dependency management (F) Installer and uninstaller (G) Consistent copyright and license statements in all source files (H) Both source and binary distributions (I) Release checklist (J) Coding standards conformance testing with enforcement (K) Test coverage testing (L) Continuous integration (M) Email address for the project.

## (4) Persistent Challenges in Research Software Development

In the long term, we expect to face three major challenges in continuing to develop khmer.

First, we need to secure continued funding for khmer software development. This will depend primarily on producing novel research, but a substantial part of our research is tied to khmer. If we can leverage our good software engineering practices to accelerate our own research while also providing value to the larger community – “better science through superior software” – then arguments for more funding will be much easier than if we simply develop khmer for others to use. This is less satisfying than getting funding for maintenance, but is a more plausible path forward than relying on maintenance grants.

Second, we must balance maintenance activities with novel research features. In the face of changing input data (due to instrument and experimental protocol changes), different expectations for output (bioinformaticians invent a new format every 5 minutes on average), competing algorithms with poor replicability, etc., we could spend 100% of our time on quality control without developing anything new. Maintenance could be a valuable community service but would not address as many student, postdoc, or faculty career incentives as doing new research. Equally, expanding our research alone would result in less reliable software. Much of our process is dedicated to walking the line between maintenance and novel research.

Third, we face many challenges in terms of recruiting software developers and researchers. Inevitably new lab members are undertrained in most of what we do, including testing, version control, good computational hygiene, data science, bioinformatics and/or the domain of biology. These are a lot of subjects to train new people in, and we have yet to establish an effective lab culture. On the converse side, of course, we expect lab graduates to be increasingly employable in both academia and biotech; moreover, the lab reputation of caring about good software has started to attract people with deeper training.

## (5) Concluding thoughts

While we are still at the early stages of the experiment, we believe we can reach some conclusions about which parts of our process have been most important. While these are anecdotes, most of our process is already standard in both industry and open source projects, so we would argue that our anecdotes bear out what is already known outside of scientific research.

First, we believe that version control and significant automated testing have both been incredibly important and are absolutely necessary for any sustained software development effort. Without version control, having multiple developers work on the same project would have been effectively impossible: all our time would have been spent on coordination issues. Even with a single developer (CTB), khmer development benefited from version history and source code comparison across versions.

Without automated testing, we would almost certainly have hesitated to make many changes, for fear of introducing regressions; this is especially important given the variance in software engineering expertise. By insisting that new code have tests associated with it, we also ensured that other developers would avoid unintentionally breaking new code they were not yet familiar with.

Second, acceptance testing has proven quite valuable for 1.0. Prior to committing to a stable command-line API, acceptance testing would most likely have been a waste of time: maintenance effort would have been needed to keep the scripts and tutorials working well together. However, now that we have committed to a stable command-line API, if the acceptance tests break it will be a bug, so there should be little maintenance burden. By committing to an Ubuntu Long Term Support release for running the acceptance tests, we can further control our maintenance costs.

Third, as we expand our development team and encourage contributions from people external to our lab, automated ways of evaluating software quality become extremely useful. Here, continuous integration, style checks, and code coverage analysis are particularly important for maintaining project stability. A formal code review by an experienced developer is the enforcement mechanism that ensures that basic requirements are met.

Our approach can be summarized thusly: we treat the development of the khmer scientific codebase as a distributed open-source project that doesn't prioritize internal over external contributions. Everyone has to meet the clearly stated expectations; un-proven experimental work by the graduate students and others are kept in separate branches until proven to be useful. Changes to the core are only introduced when necessary and not any sooner (as in the style of agile development). This allows us to balance the two purposes of khmer: as a production codebase and as a foundation for research.

One important caveat is that we don't yet know how well any of this is going to work! Our chief goals are to enable further research with khmer and maintain existing functionality, all while our developer base expands and/or turns over. We hope and believe that our approaches will let us do this, but we need a longer baseline of observations to find out.
